# Prospective evaluation of PI-RADSv2.1 using multiparametric and biparametric MRI for detecting clinically significant prostate cancer based on MRI/US fusion-guided biopsy

**DOI:** 10.1007/s11604-024-01675-4

**Published:** 2024-10-16

**Authors:** Naohiro Yamaya, Koichiro Kimura, Ryota Ichikawa, Masaaki Kawanishi, Yusuke Kawasaki, Subaru Higuchi, Kenichi Fukui, Junichi Tsuchiya, Masaki Kobayashi, Soichiro Yoshida, Yasuhisa Fujii, Ukihide Tateishi

**Affiliations:** 1 Department of Diagnostic Radiology, Tokyo Medical and Dental University, 1-5-45 Yushima, Bunkyo-ku, Tokyo, 113–8510 Japan; 2 Department of Radiology, Kameda Medical Center, Chiba, Japan; 3 Department of Diagnostic Imaging, Cancer Institute Hospital of JFCR, Tokyo, Japan; 4 Department of Diagnostic Radiology, JA Toride Medical Center, Ibaraki, Japan; 5 Department of Radiology, Asahi General Hospital, Chiba, Japan; 6 Department of Radiology, Soka City Hospital, Saitama, Japan; 7 Department of Urology, Tokyo Medical and Dental University, Tokyo, Japan

**Keywords:** Prostate Cancer, Prostate Imaging-Reporting and Data System version 2.1, Multiparametric MRI, Biparametric MRI, MRI/US-fusion biopsy

## Abstract

**Purpose:**

To evaluate the cancer detection rates for each category of Prostate Imaging-Reporting and Data System version 2.1 (PI-RADSv2.1) using multiparametric magnetic resonance imaging (mpMRI) and biparametric MRI (bpMRI) based on MRI/ultrasound (US)-fusion biopsy.

**Materials and methods:**

This prospective study included participants who underwent mpMRI or bpMRI with a PI-RADSv2.1 interpretation and subsequently received MRI/US-fusion biopsy between August 2022 and December 2023. The lesion-based detection rates of clinically significant prostate cancer (csPCa) in each PI-RADSv2.1 category and the correlation between PI-RADSv2.1 categories and International Society of Urological Pathology (ISUP) grade groups were analyzed. The diagnostic performance of PI-RADSv2.1 in predicting csPCa was evaluated, and diagnostic performance of mpMRI and bpMRI was compared using cut-offs, with PI-RADSv2.1 categories ≥ 3 or ≥ 4 defined as positive.

**Results:**

A total of 247 lesions from 216 participants were included in this study. A total of 157 patients underwent mpMRI and the remaining 59 underwent bpMRI. The csPCa detection rates for each PI-RADSv2.1 category of mpMRI and bpMRI were as follows: category 1, 0% (0/11); 2, 13% (3/23); 3, 16% (5/31); 4, 60% (43/72); 5, 65% (26/40), in mpMRI; category 1, 0% (0/4); 2, 33% (1/3); 3, 25% (3/12); 4, 61% (19/31); 5, 75% (15/20) in bpMRI. PI-RADSv2.1 categories were significantly positively associated with csPCa detection rates in both mpMRI and bpMRI (*p* < 0.0001 and *p* = 0.00048, respectively). PI-RADSv2.1 categories correlated with ISUP grade groups for mpMRI and bpMRI (*p* < 0.0001 for both). There were no significant differences in the detection rates between mpMRI and bpMRI for PI-RADS v2.1 positive and negative lesions.

**Conclusion:**

PI-RADSv2.1 using mpMRI and bpMRI could stratify the risk of csPCa, and the csPCa detection rate of bpMRI was compatible with that of mpMRI using cut-offs of PI-RADSv2.1 categories ≥ 3 or ≥ 4.

## Introduction

Prostate cancer (PCa) is the second most common cancer in men and fourth most common malignancy considering both sexes. Most PCa cases in the United States grow slowly and showed a 5-year relative survival rate of 97%. Despite a good prognosis, due to the large number of patients with PCa, the number of deaths has reached 396,792, with PCa being the fifth leading cause of cancer related mortality among men in 2022 [1]. The American Cancer Society recommends that asymptomatic males, who are expected to have a 10-year life expectancy, be offered the opportunity to make an informed decision with their healthcare provider about PCa screening with prostate-specific antigen (PSA) testing [2].

Multiparametric magnetic resonance imaging (mpMRI) undoubtedly affects the efficient detection of clinically significant PCa (csPCa) in patients with elevated PSA levels or abnormal digital rectal examinations [3]. The Prostate Imaging-Reporting and Data System version 2.1 (PI-RADSv2.1), released in 2019, is a guide to standardize mpMRI examination and interpretation of prostate MRI to detect csPCa using a 5-point Likert scale [4]. The latest systematic review and meta-analysis showed that the pooled csPCa detection rates of PI-RADSv2.1 in the lesion-level analysis were 2% for category 1, 4% for category 2, 20% for category 3, 52% for category 4, and 89% for category 5 [5]. However, these results were mainly derived from retrospective studies and a few prospective studies. To the best of our knowledge, only two prospective studies have demonstrated the risk stratification of csPCa using PI-RADSv2.1, and these studies were conducted by highly-experienced readers in limited institutions [6, 7]. Furthermore, no prospective data exist on the csPCa detection rates of PI-RADSv2.1 in a Japanese cohort. It is well known that the cancer detection rate using the previous version of PI-RADS (v2.0) varies depending on the experience of the evaluator and the institution [8]. Thus, real-world data on the diagnostic performance of PI-RADSv2.1 are insufficient and premature. In addition, biparametric MRI (bpMRI), which does not require dynamic contrast-enhanced (DCE) imaging, is often performed in clinical practice for various reasons that impair the administration of gadolinium (Gd)-based contrast agents. A meta-analysis of head-to-head comparisons of bpMRI and mpMRI using PI-RADSv1.0 or v2.0 showed equivalent performances in terms of pooled sensitivity and specificity [9]. However, the PI-RADS committee is unable to conclude its utility or provide a statement on bpMRI. Therefore, the diagnostic performance of bpMRI following the PI-RADSv2.1 scheme has not been established, and the feasibility of bpMRI in all prostate MRI remains controversial.

In the last decade, MRI/ultrasound (US)-fusion biopsy has emerged as a solution to the problems associated with conventional transrectal US-guided cognitive biopsy, including misregistration for biopsy, which leads to the underestimation of csPCa and an increase in unnecessary biopsies [10]. MRI/US-fusion biopsy can achieve more accurate localization of target lesions and has been reported to have superior detectability to standard transrectal US-guided biopsy in patients at clinical risk for PCa [11–13].

Since the real-world data of the performance of PI-RADSv2.1 in both mpMRI and bpMRI can be accurately evaluated using MRI/US-fusion biopsy, this study conducted a prospective evaluation of the cancer detection rates for each PI-RADSv2.1 category with MRI/US fusion-guided biopsy.

## Materials and methods

### Study participants

**Fig. 1:**
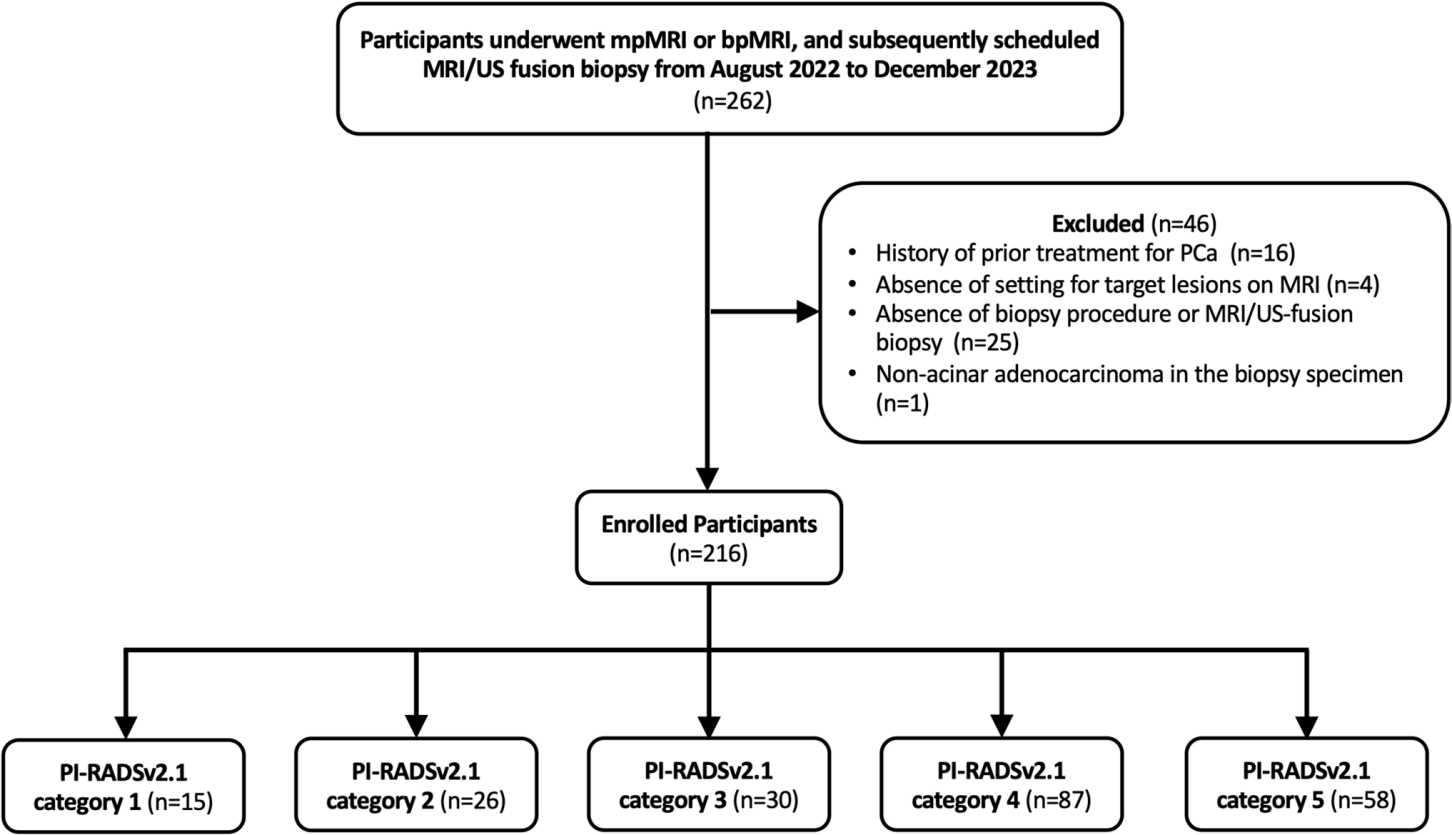
Flowchart of the study participants. *mpMRI*, multiparametric MRI; *bpMRI*, biparametric MRI; *PCa*, prostate cancer; *PI-RADS*, Prostate Imaging-Reporting and Data System

This prospective study was approved by the Institutional Review Board of Tokyo Medical and Dental University Hospital (M2000-2130-01), and written informed consent was obtained from each participant. Between August 2022 and December 2023, patients who underwent specific prostate mpMRI or bpMRI and were scheduled for a subsequent systematic 12-core MRI/US fusion-guided biopsy were prospectively and consecutively registered. The decisions regarding whether to perform mpMRI or bpMRI were made by several urologists in charge of the patients based on each patient’s history and clinical data. The decision to recommend prostate biopsy to a patient was made by integrating all clinical data, including initial reports of prostate mpMRI or bpMRI interpreted by unspecified diagnostic radiologists. The exclusion criteria were as follows: (a) history of prior PCa treatment, (b) determined absence of target lesions on MRI by a board-certified radiologist, (c) absence of a biopsy procedure or MRI/US fusion-guided biopsy, and (d) non-acinar adenocarcinoma in the biopsy specimen. All PCa-suspected participants who met the study criteria were considered eligible and included in the final study sample, and the selection process is shown in Fig. 1. Patients’ clinical data were extracted from institutional electronic medical records.

### Prostate MRI protocol and image analysis

All patients were examined using a 3-T MR scanner (Ingenia CX, Philips Healthcare, Best, the Netherlands) with a 32-channel sensitivity-encoding body coil. The mpMRI protocol included T2-weighted imaging (T2WI), high–*b* value diffusion-weighted imaging (DWI), and DCE imaging, whereas the bpMRI protocol included minimal T2WI and high–*b* value DWI. Details of the image acquisition parameters and Gd-based contrast agent used are provided in Table 1. Briefly, a volume isotropic turbo spin-echo (VISTA) 3D T2WI sequence was performed to obtain three-dimensional images. The multiplanar reconstructed images were generated using the VISTA dataset. DWI was performed using three different *b* values (0, 1000, and 2000s/mm^2^), and apparent diffusion coefficient (ADC) maps were acquired. DCE imaging was performed using a fat-suppressed 3D volumetric spoiled gradient-echo sequence before and after the intravenous injection of a Gd-based contrast agent (temporal resolution, 11.7 s).

**Table 1:** MRI scan protocols

	n = 216 (mpMRI n = 157; bpMRI n = 59)		
	3D-T2WI	DWI	DCE imaging
Type of sequence	3D-FSE	SS-EPI	3D-GRE
Orientation	Axial	Axial	Axial
TR/TE (ms)	1500/200	5000/80	3.1/1.6
Flip angle (degree)	90	90	12
FOV (cm)	30	30	30
Matrix	255×255	128×112	256×180
Slice thickness (mm)	1.6	4	4.4
Slice gap (mm)	0	0.4	0
Number of excitations	1	6, 6, 14	1
*b*-value (s/mm^2^)	–	0, 1000, 2000	–
Gd-based contrast agent	–	–	Gadobutrol
Dose (mL/kg), flow rate (mL/s)	–	–	0.1, 1.5
Timing of DCE image after injection (s)	–	–	11.7, 23.4, 35.1, 46.8, 58.5, 70.2, 81.9, 93.6, 105.3

Gd-based contrast agent: gadobutrol, Gadovist, Bayer Schering Pharma

*mpMRI* multiparametric MRI, *bpMRI* biparametric MRI, *3D-T2WI* three-dimensional T2-weighted imaging, *DWI* diffusion-weighted imaging, *DCE* dynamic contrast-enhanced, *3D-FSE* three-dimensional fast spin-echo, *SS-EPI* single-shot echo planar imaging, *3D-GRE* three-dimensional gradient-echo, *TR/TE* repetition time/echo time, *FOV* field of view, *Gd* Gadolinium

All pre-biopsy mpMRIs or bpMRIs were centrally and prospectively evaluated by a urogenital radiologist (K.K.) with six years of experience in prostate imaging. Each intraprostatic lesion found on MRI was scored based on the PI-RADSv2.1 criteria and reported to the urologist as the target of MRI/US fusion-guided biopsy.

### Biopsy procedure and pathologic assessment

All MRIs were imported into a commercial biopsy preparation system (DynaCAD, Invivo/Philips, Gainesville, FL, USA) for biopsy preparation, and the target lesions were volumetrically contoured on T2WI with DWI, ADC map, and DCE. MRI/US fusion-guided biopsy was executed using a commercial biopsy system (UroNav, Invivo/Philips, Gainesville, FL, USA), and 3-core were obtained for each lesion before systematic 12-core biopsy. When a patient had multiple target lesions, which were PI-RADSv2.1 categories ≥ 3, MRI/US fusion-guided biopsy was carried out for at most three lesions in decreasing order of PI-RADSv2.1 category and size. In this study, patients who were evaluated by a certified radiologist for non-cancerous MRI findings (such as PI-RADSv2.1 category 1 or 2 for hyperplastic nodule or atypical nodule, respectively, on transitional zone [TZ] and PI-RADSv2.1 category 2 for inflammatory changes on peripheral zone [PZ]) were also included and underwent systematic biopsy and MRI/US fusion-guided biopsy for the lesions of PI-RADSv2.1 category ≤ 2. This was because some of those patients were referred to our institution for a thorough examination due to abnormally high PSA levels on prostate MRI at previous hospitals, indicating lesions in the PI-RADSv2.1 category ≥ 3. Biopsies were performed using an 18-gauge biopsy gun with a 19 mm specimen size. All preparation and biopsy procedures were performed by several urologists, each with at least three years of experience in systematic and MRI/US fusion-guided biopsy [14].

The International Society of Urologic Pathology (ISUP) consensus conference of the Gleason grade group was used to classify the biopsy specimens by dedicated pathologists who were blinded to all the MRI findings. To summarize this ISUP grade group, 1 has the same rank as a Gleason score of 3 + 3; 2, Gleason 3 + 4; 3, Gleason 4 + 3; 4, Gleason 4 + 4; 5, a Gleason sum of 9 or 10. In this study, ISUP grade group ≥ 2 was defined as csPCa. Because the purpose of this study was to evaluate the cancer detection rates for each PI-RADSv2.1 category with MRI/US fusion-guided biopsy on mpMRI and bpMRI, and target biopsy is more accurate than systematic biopsy, which could not match the specific lesions on MRI, we did not consider the pathology results of systematic biopsy specimens in the present study, as in past prospective studies.

### Statistical analysis

Pre-estimation of the sample size was not performed in the present study. All patients who met the inclusion criteria in the given period were included in the study cohort. A lesion-based analysis was conducted to determine the proportion of csPCa in each PI-RADSv2.1 category. All statistical analyses were performed using R version 4.0.3 (R Foundation for Statistical Computing; https://www.r-project.org/) and the JMP 14.2 statistical software (SAS Institute Inc., Cary, NC, USA). Categorical clinical data including the PI-RADSv2.1 category are presented as numbers (percentages) and compared using Fisher’s exact test, whereas continuous clinical variables are expressed as the median (interquartile range [IQR]) and compared using the Wilcoxon rank-sum test. The Cochran–Armitage trend test was used to evaluate the association between the PI-RADSv2.1 category and cancer detection rates. Kendall tau-b statistics were used to assess the correlation between the PI-RADSv2.1 categories and ISUP grade groups. The diagnostic performance of the PI-RADSv2.1 category for predicting csPCa was evaluated by calculating the sensitivity, specificity, accuracy, positive predictive value (PPV), and negative predictive value (NPV) using cut-offs PI-RADSv2.1 category ≥ 3 or ≥ 4 defined as positive. A *p*-value < 0.05 was considered statistically significant.

## Results

### Characteristics of the participants

Between August 2022 and December 2023, 262 patients underwent MRI/US fusion-guided biopsy. After excluding 46 patients (46 lesions), 247 lesions in 216 patients were included in the analysis (Fig. 1). The patients’ demographic characteristics are shown in Table 2. A total of 157 patients (73%) underwent mpMRI and the remaining 59 (27%) underwent bpMRI. The characteristics of the participants at baseline were similar in the mpMRI and bpMRI groups except for the estimated glomerular filtration rate (eGFR) (64.4 mL/min/1.73 m^2^ vs. 54.2 mL/min/1.73 m^2^, *p* < 0.0001), history of asthma (0/157, 0% vs. 4/59, 7%, *p* = 0.0052), and allergy to Gd-based contrast agents (0/157, 0% vs. 5/59, 8%, *p* = 0.0013). The median patient age was 71 years (IQR 64–75 years). The median PSA level was 8.2 ng/mL (IQR 5.4–12.2). Fifty-three patients (25%) had a prior prostate biopsy, and twelve (6%) were already diagnosed with PCa and were on active surveillance. Ninty-nine lesions (40%) were in the TZ and 148 lesions (60%) were in the PZ.

**Table 2:** Demographic characteristics of the eligible patients

		Total patients (n = 216)	mpMRI (n = 157)	bpMRI (n = 59)	*p* value
Age, years^*^		71 (64–75)	70 (64–74)	71 (65–76)	0.11
Prior biopsy	None	163 (75)	119 (76)	44 (74)	0.89
	Yes, negative	41 (19)	30 (19)	11 (19)	
	Yes, positive	12 (6)	8 (5)	4 (7)	
PSA level (ng/mL)^*^		8.2 (5.4–12.2)	8.3 (5.4–12.4)	7.2 (5.2–10.8)	0.28
eGFR (mL/min/1.73m^2^)^*^		62.1 (54.2–69.3)	64.4 (57.4–70.3)	54.2 (44.4–65.5)	< 0.0001
History of asthma	Yes	4 (2)	0 (0)	4 (7)	0.0052
Allergy to Gd-based contrast agents	Yes	5 (2)	0 (0)	5 (8)	0.0013
Total number of biopsy lesions		247	177 (72)	70 (28)	
Total detection rate of PCa^†^		60 (149/247)	59 (104/177)	64 (45/70)	0.47
Total detection rate of csPCa^†^		47 (115/247)	44 (77/177)	54 (38/70)	0.16
Location of lesions	TZ	99 (40)	77 (44)	22 (31)	0.086
	PZ	148 (60)	100 (56)	48 (69)	
Highest PI-RADSv2.1 category	1	15 (7)	11 (7)	4 (7)	0.27
	2	26 (12)	23 (15)	3 (5)	
	3	30 (14)	23 (15)	7 (12)	
	4	87 (40)	62 (39)	25 (42)	
	5	58 (27)	38 (24)	20 (34)	
Highest ISUP grade group	Benign	85 (39)	65 (41)	20 (34)	0.28
	1	26 (12)	20 (13)	6 (10)	
	2	43 (20)	32 (20)	11 (19)	
	3	24 (11)	13 (8)	11 (19)	
	4	26 (12)	20 (13)	6 (10)	
	5	12 (6)	7 (4)	5 (8)	

Unless otherwise mentioned, values in parentheses are percentages of cases

*Median (interquartile range)

^†^Percentage

*mpMRI* multiparametric MRI, *bpMRI* biparametric MRI, *PSA* prostate-specific antigen, *eGFR* estimated glomerular filtration rate, *Gd* Gadolinium, *PCa* prostate cancer, *csPCa* clinically significant prostate cancer, *PI-RADS* Prostate Imaging Reporting and Date System, *ISUP* International Society of Urological Pathology

### Association between PI-RADSv2.1 categories and cancer detection rate

**Fig. 2:**
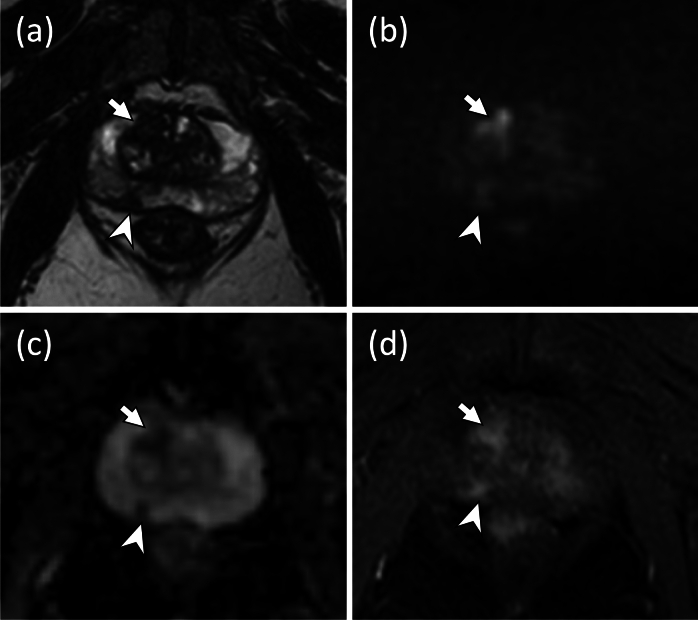
Multiparametric MRI of a 72-year-old man with 6.4 ng/mL PSA level. **a** Axial T2WI showing a hypointense mass (< 15 mm) in the right midgland peripheral zone posteromedial (arrowhead). **b, c** Axial DWI (*b* = 2000s/mm^2^) (**b**) revealing the mass as focal mild hyperintensities (arrowhead), and axial ADC map showing a markedly hypointense mass (arrowhead) (**c**). **d** Axial DCE image showing an early enhancement in the location corresponding to the same mass in **a**–**c** (arrowhead), which suggests that an overall PI-RADSv2.1 category 4 (consistent with a T2WI score of 4, a DWI score of 3, and a DCE positive) can be obtained. MRI/US fusion-guided biopsy revealed csPCa with an ISUP grade group 3. In addition, arrows in a–d indicate PI-RADSv2.1 category 3 (consistent with a T2WI score of 2, a DWI score of 4, and a DCE positive) lesions visible in the right midgland transition zone anterior. MRI/US fusion-guided biopsy showed no evidence of cancerous tissue in this lesion. *PSA,* prostate-specific antigen; *T2WI*, T2-weighted imaging; *DWI*, diffusion-weighted imaging; *ADC*, apparent diffusion coefficient; *DCE*, dynamic contrast-enhanced; *PI-RADS*, Prostate Imaging-Reporting and Data System; *csPCa*, clinically significant prostate cancer; *ISUP*, International Society of Urological Pathology

**Fig. 3:**
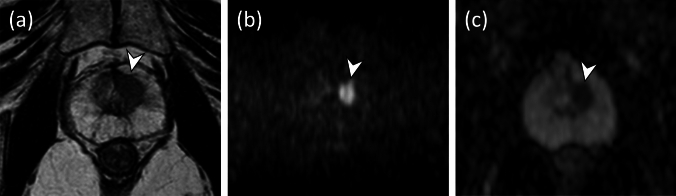
Biparametric MRI of a 75-year-old man with 9.8 ng/mL PSA level. **a** Axial T2WI showing a homogeneous hypointense mass (< 15 mm) in the left midgland transition zone anterior (arrowhead). **b, c** Axial DWI (*b* = 2000s/mm^2^) (**b**) revealing the mass as markedly hyperintense (arrowhead), axial ADC map (**c**) showing a markedly hypointense mass (arrowhead), which suggests that an overall PI-RADS category 4 (consistent with of a T2WI score of 4 and a DWI score of 4) can be obtained. MRI/US fusion-guided biopsy revealed csPCa with an ISUP grade group 3. *PSA,* prostate-specific antigen; *T2WI*, T2-weighted imaging; *DWI*, diffusion-weighted imaging; *ADC*, apparent diffusion coefficient; *PI-RADS*, Prostate Imaging-Reporting and Data System; *csPCa*, clinically significant prostate cancer; *ISUP*, International Society of Urological Pathology

At the lesion level, MRI/US fusion-guided biopsy detected PCa and csPCa in 60% (149/247) and 47% (115/247) lesions, respectively. The number of each PI-RADSv2.1 category was as follows: category 1, 15; category 2, 26; category 3, 43; category 4, 103; category 5, 60. Table 3 presents the cancer detection rates for each PI-RADSv2.1 category of mp-MRI and bp-MRI. The csPCa detection rates for each PI-RADSv2.1 category of mpMRI and bpMRI were as follows: category 1, 0% (0/11); category 2, 13% (3/23); category 3, 16% (5/31); category 4, 60% (43/72); category 5, 65% (26/40), in mpMRI; category 1, 0% (0/4); category 2, 33% (1/3); category 3, 25% (3/12); category 4, 61% (19/31); category 5, 75% (15/20) in bpMRI. The detection rates of PCa and csPCa tended to increase as the PI-RADSv2.1 category increased, and the Cochran–Armitage test showed that the PI-RADSv2.1 category was significantly positively associated with the detection rates of PCa and csPCa in both mpMRI (*p* < 0.0001 for both) and bpMRI (*p* < 0.0001 and *p* = 0.00048, respectively). Figures 2 and 3 show representative patients with pathologically confirmed csPCa with PI-RADSv2.1 category 4 in mpMRI and bpMRI, respectively.

**Table 3:** Cancer detection rates for each PI-RADSv2.1 category of mpMRI and bpMRI

		PI-RADSv2.1 category					
		1	2	3	4	5	*p* value^*^
mpMRI	PCa	18 (2/11)	17 (4/23)	45 (14/31)	72 (52/72)	80 (32/40)	< 0.0001
	csPCa	0 (0/11)	13 (3/23)	16 (5/31)	60 (43/72)	65 (26/40)	< 0.0001
bpMRI	PCa	0 (0/4)	33 (1/3)	33 (4/12)	74 (23/31)	85 (17/20)	< 0.0001
	csPCa	0 (0/4)	33 (1/3)	25 (3/12)	61 (19/31)	75 (15/20)	0.00048

*The Cochran–Armitage test was performed

*PI-RADS* Prostate Imaging-Reporting and Data System, *mpMRI* multiparametric MRI, *bpMRI* biparametric MRI, *PCa* prostate cancer, *csPCa* clinically significant prostate cancer

### Correlation between PI-RADSv2.1 categories and ISUP grade groups

**Fig. 4:**
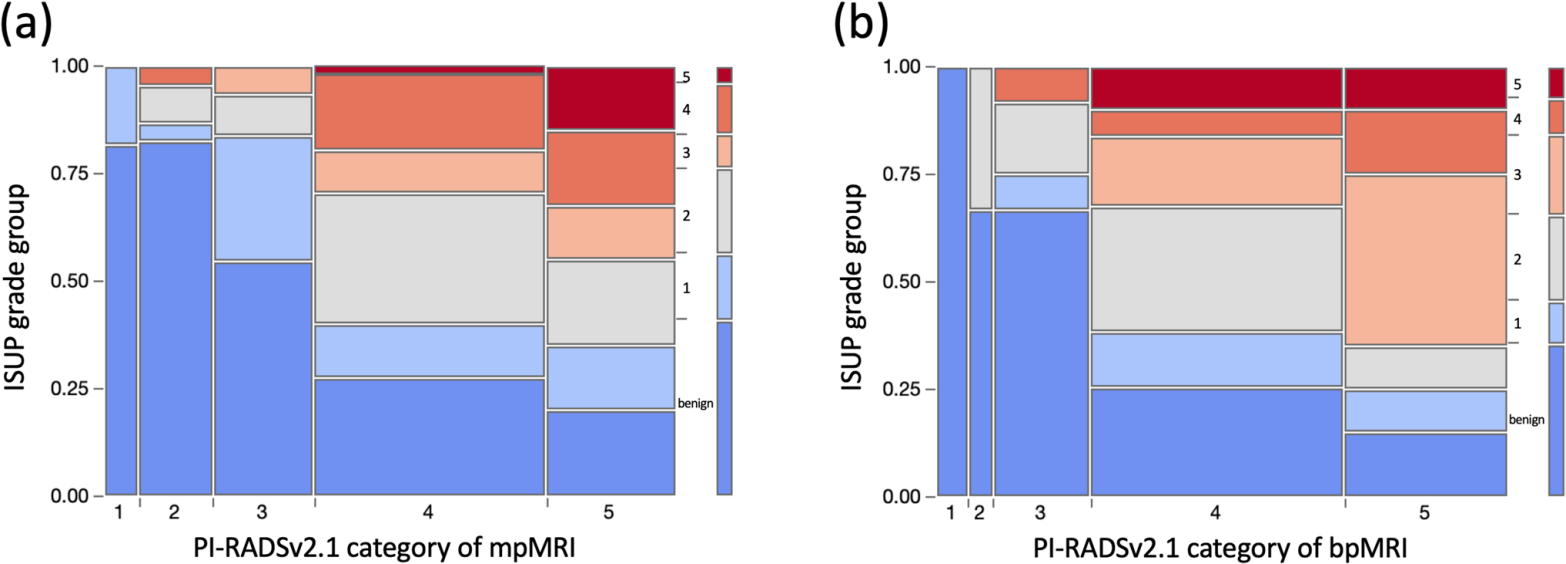
Mosaic plots of PI-RADSv2.1 categories and ISUP grade groups in both mpMRI (**a**) and bpMRI (**b**). *PI-RADS*, Prostate Imaging Reporting and Data System; *ISUP*, International Society of Urological Pathology; *mpMRI*, multiparametric MRI; *bpMRI*, biparametric MRI; *PCa*, prostate cancer

Figure 4 shows mosaic plots of the PI-RADSv2.1 categories and ISUP grade groups in both mpMRI and bpMRI. The Kendall tau-b rank correlation between PI-RADSv2.1 categories and ISUP grade groups in mpMRI and bpMRI for all prostate lesions were 0.42 and 0.42, respectively (both, *p* < 0.0001).

### Diagnostic performance of mpMRI and bpMRI

Table 4 summarizes the sensitivity, specificity, accuracy, PPV, and NPV for csPCa diagnosis when PI-RADSv2.1 categories ≥ 3 or ≥ 4 were considered positive. When the PI-RADS cut-off of mpMRI was set at ≤ 2 (n = 34)/ ≥ 3 (n = 143), the sensitivity, specificity, and accuracy were 0.96 (95% confidence interval [CI]: 0.89–0.99), 0.31 (95% CI: 0.22–0.41), and 0.59 (95% CI: 0.52–0.67), respectively. When the PI-RADS cut-off of bpMRI was set at ≤ 2 (n = 7)/ ≥ 3 (n = 63), the sensitivity, specificity, and accuracy were 0.97 (95% CI: 0.86–1.00), 0.19 (95% CI: 0.07–0.36), and 0.61 (95% CI: 0.49–0.73), respectively. When the PI-RADS cut-off of mpMRI was set at ≤ 3 (n = 65)/ ≥ 4 (n = 112), the sensitivity, specificity, and accuracy were 0.90 (95% CI: 0.81–0.95), 0.57 (95% CI: 0.47–0.70), and 0.71 (95% CI: 0.64–0.78), respectively. When the PI-RADS cut-off of bpMRI was set at ≤ 3 (n = 19)/ ≥ 4 (n = 51), the sensitivity, specificity, and accuracy were 0.90 (95% CI: 0.75–0.97), 0.47 (95% CI: 0.29–0.65), and 0.70 (95% CI: 0.58–0.80), respectively. There was no significant difference in the csPCa detection rate between mpMRI and bpMRI for PI-RADS-negative and positive lesions at either cut-off.

**Table 4:** Sensitivity, specificity, accuracy, PPV, and NPV for csPCa diagnosis when PI-RADS score ≥ 3 and ≥ 4 defined as positive

		Sensitivity (95% CI)	Specificity (95% CI)	Accuracy (95% CI)	PPV (95% CI)	NPV (95% CI)	*p* value^*^	*p* value^†^
Cut-off PI-RADSv2.1 category ≥ 3	mpMRI	0.96 (0.89–0.99)	0.31 (0.22–0.41)	0.59 (0.52–0.67)	0.52 (0.43–0.60)	0.91 (0.76–0.98)	0.37	0.54
	bpMRI	0.97 (0.86–1.00)	0.19 (0.07–0.36)	0.61 (0.49–0.73)	0.59 (0.46–0.71)	0.86 (0.42–1.00)		
Cut-off PI-RADS v2.1 category ≥ 4	mpMRI	0.90 (0.81–0.95)	0.57 (0.47–0.70)	0.71 (0.64–0.78)	0.62 (0.52–0.71)	0.88 (0.77–0.95)	0.60	0.46
	bpMRI	0.90 (0.75–0.97)	0.47 (0.29–0.65)	0.70 (0.58–0.80)	0.67 (0.52–0.79)	0.79 (0.54–0.94)		

*p values are comparisons of detection rate between mpMRI and bpMRI in lesions with PI-RADSv2.1 positive lesions (score ≥ 3 and ≥ 4, respectively), and are calculated using Fisher’s exact test

^†^*p* values are comparisons of detection rate between mpMRI and bpMRI in lesions with PI-RADS v2.1 negative lesions (score ≤ 2 and ≤ 3, respectively) and are calculated using Fisher’s exact test

*PPV* positive predictive value, *NPV* negative predictive value, *PI-RADS* Prostate Imaging-Reporting and Data System, *mpMRI* multiparametric MRI, *bpMRI* biparametric MRI, *CI* confidence interval

## Discussion

Real-world evidence on the diagnostic performance of PI-RADSv2.1 using both mpMRI and bpMRI is insufficient because prospective design studies are scarce, and the results of conventional US-guided cognitive biopsies are uncertain. In the present prospective study, we confirmed that PI-RADS v2.1 in mpMRI and bpMRI could stratify prostate lesions according to their risk of csPCa, based on specimens obtained by MRI/US fusion-guided biopsy in a Japanese cohort. Although the number of lesions evaluated using bpMRI was small, the csPCa detection rate of bpMRI was comparable to that of mpMRI using PI-RADSv2.1 categories ≥ 3 or ≥ 4. Our data help solidify the evidence for the feasibility and utility of PI-RADSv2.1 using mpMRI for PCa-suspected patients and bpMRI for patients who cannot be administered a Gd-based contrast agent in clinical practice.

As observed in two studies using prospective cohorts [6, 7], the current study also showed that a higher PI-RADSv2.1 category increased the detectability of csPCa in mpMRI. The detection rate of PI-RADSv2.1 category 4 of mpMRI (60%, 43/72) and bpMRI (61%, 19/31) was relatively higher than that in previous studies (37–44%). The higher detection rate of PI-RADSv2.1 category 4 in the presented data might result from the difference in the study cohort or less years of experience of the evaluator [15].

Understanding the differences between prospective and retrospective studies on csPCa detection rates in each category is necessary for credible use of the PI-RADS system in clinical practice. Interestingly, the range of lesion-based csPCa detection rates for PI-RADSv2.1 category 5 of mpMRI in current and previous prospective studies was 65–80% [6, 7], somewhat lower than the 89% reported in a meta-analysis mainly composed of retrospective studies [5]. This indicates that the csPCa detection rate in retrospective studies might overestimate category 5 owing to problems with the relative inaccuracy of the evaluation method in the retrospective design. In retrospective studies, where analyzed groups were derived from patients who have undergone biopsies or surgeries to match pathological outcomes with lesions identified on MRI, there was a possibility that non-cancerous lesions, which might have been rated as PI-RADSv2.1 category 5 in a prospective setting, were excluded from the analysis due to escaping biopsy. As a result, the detection rate of PI-RADSv2.1 category 5 lesions in a retrospective design may be higher than that observed in a prospective design. It could be considered that more experienced evaluators might be less likely to erroneously judge non-cancerous lesions as PI-RADSv2.1 category 5 in a prospective setting. Further evidence from prospective studies reviewed by radiologists with different experiences is needed to establish the csPCa risk rate of PI-RADSv2.1 in category 5.

Although the total number of lesions evaluated by bpMRI was small, a significant trend was observed where higher PI-RADSv2.1 categories in bpMRI increased the detectability of csPCa. This result aligns closely with a previous study that demonstrated higher csPCa detection rates in higher PI-RADSv2.0 categories using bpMRI [16]. The only exception was the reversed trend in detection rates observed in PI-RADSv2.1 categories 2 (33%, 1/3) and 3 (25%, 3/12) in bpMRI, which could be attributed to the small sample size in category 2. In addition, although concerns regarding being underpowered for the detection of difference still remain, no significant differences in detection rate between mpMRI and bpMRI in lesions with PI-RADS v2.1 positive (score ≥ 3 or ≥ 4) and negative (score ≤ 2 or ≤ 3) were found. If the detection rate remains consistent, bpMRI can be considered a more efficient test than mpMRI because of its shorter testing time and lower performing cost [17]. Additionally, even if the rate of side effects to Gd-based contrast agents is low, patients would not be exposed to unnecessary risks when prostate MRI can omit DCE imaging. However, it should be noted that even with our positive bpMRI results, adopting the bpMRI approach was not perfectly acceptable. DCE imaging may prevent radiologists with less experience from missing findings, such as lesions located at the apex of the prostate and TZ lesions that can be first noticed by early enhancement. Furthermore, DCE imaging contributes to locoregional staging decisions related to treatment strategies [18]. However, in actual clinical practice, it is also true that urologists order to perform bpMRI for a variety of patient-specific reasons, even if mpMRI has advantages. In our cohort, the types of MRI were determined by the attending urologists. Patients with bpMRI have significantly lower renal function than those with mpMRI, and the patients with a history of asthma and allergy to Gd-based contrast agents underwent bpMRI. The current results showed no problems with the detection rate of csPCa using bpMRI in specific patients; however, it was premature to apply bpMRI in all patients. Two ongoing prospective multicenter clinical trials have evaluated whether bpMRI is non-inferior to mpMRI in the detection of csPCa [19, 20]. These clinical trials may provide evidence supporting the omission of Gd-based contrast agents in prostate MRI.

In this study, the likelihood of malignancy increased with increasing PI-RADSv2.1 category on pre-biopsy MRI. PI-RADSv2.1 categories and ISUP grade groups showed a correlation with Kendall tau-b rank statistics (0.42 for both), which was comparable between mpMRI and bpMRI. Walker et al. also reported a Kendall tau-b rank correlation of 0.50 between PI-RADSv2.1 categories and ISUP grade groups in their prospective cohort [6]. The increase in csPCa detection rate and malignancy with increasing categories, both in mpMRI and bpMRI, showed that the PI-RADS achieved its objective, which is the development of assessment categories that summarize levels of suspicion or risk and can be used to select patients for biopsies and beyond management (e.g., immediate interventions or observation strategy).

Our study has several limitations. First, this was a single-center study with a relatively small cohort, and all mpMRI and bpMRI scans were interpreted by a single urogenital radiologist owing to its prospective design. Although the inter-reader reliability of mpMRI and bpMRI using PI-RADS v2.1 was reported to be comparable in a retrospective study [21], further prospective evidence is needed to confirm its reproducibility. Second, mpMRI or bpMRI assignment was left to the attending physician depending on the individual patient’s situation. Except for the factors that prevented the administration of Gd-based contrast agents, there was no significant association between the representative information for suspected PCa, such as PSA, and the availability of performing DCE imaging. However, the patient’s other factors that cannot be accurately analyzed (e.g. subjectivity of the attending physician in the digital rectal exam, fluctuations in PSA levels) may have influenced how the prostate MRI was planned. As a result, there might be an unrecognizable selection bias, leading to the preferential assignment of patients whose PCa lesions were more easily depicted on MRI for bpMRI. This could result in an overestimation of the detection rate of csPCa in bpMRI. Third, because 3D volume data of the prostate were essential for conducting the MRI/US fusion-guided biopsies, T2WI was obtained using a 3D fast spin-echo sequence in the current study, not the 2D fast spin-echo sequence that is recommended for PI-RADSv2.1 assessment. Several published studies have reported that the PI-RADS category using 3D T2WI showed a diagnostic performance comparable to that of the usual PI-RADS category using 2D T2WI [22, 23]. In practice, though 3D T2WI can depict high-resolution imaging, its signal-to-noise ratio and contrast-to-noise ratio tend to be lower than those of 2D T2WI. This difference might have made it somewhat more difficult to detect csPCa lesions, particularly in the TZ, in our study. Finally, only the results of MRI/US fusion-guided biopsies were considered, and systematic biopsies in some participants may have demonstrated the diagnosis of csPCa with negative MRI/US fusion-guided biopsies. However, the interpretation of the discrepancy in results between target and systematic biopsies was complicated, and we decided to employ only the results of target biopsies to clarify the correlation of csPCa detection rates across different PI-RADSv2.1 categories of both mpMRI and bpMRI.

In conclusion, our prospective studies demonstrated that PI-RADSv2.1 using mpMRI and bpMRI could stratify the risk of csPCa, and csPCa detection rate of bpMRI in specific patients was comparable to that of mpMRI using cut-offs of PI-RADSv2.1 categories ≥ 3 or ≥ 4. PI-RADSv2.1 category 5 had lower csPCa detection rates than previous prospective studies or systemic reviews. Our findings may help PI-RADS users to properly understand the advantages and limitations of PI-RADS’s diagnostic capabilities and enable the accurate use of PI-RADSv2.1 in bpMRI with specific patients, in addition to mpMRI in clinical practice.
